# Association between benzodiazepine receptor agonist use and mortality in patients hospitalised for COVID-19: a multicentre observational study

**DOI:** 10.1017/S2045796021000743

**Published:** 2022-03-30

**Authors:** N. Hoertel, M. Sánchez-Rico, E. Gulbins, J. Kornhuber, R. Vernet, N. Beeker, A. Neuraz, C. Blanco, M. Olfson, G. Airagnes, C. Lemogne, J. M. Alvarado, M. Arnaout, C. Cougoule, P. Meneton, F. Limosin

**Affiliations:** 1 Département de Psychiatrie, Assistance Publique-Hôpitaux de Paris (AP-HP), Hôpital Corentin-Celton, DMU Psychiatrie et Addictologie, Issy-les-Moulineaux, France; 2 Université de Paris, Paris, France; 3 INSERM, Institut de Psychiatrie et Neurosciences de Paris (IPNP), UMR_S1266, Paris, France; 4 Department of Psychobiology & Behavioural Sciences Methods, Faculty of Psychology, Universidad Complutense de Madrid, Campus de Somosaguas, Pozuelo de Alarcon, Spain; 5 Institute for Molecular Biology, University Medicine Essen, University of Duisburg-Essen, Essen, Germany; 6 Department of Psychiatry and Psychotherapy, University Hospital Friedrich-Alexander-University of Erlangen-Nuremberg, Erlangen, Germany; 7 Medical Informatics, Biostatistics and Public Health Department, AP-HP, Centre-Université de Paris, Hôpital Européen Georges Pompidou, F-75015 Paris, France; 8 Assistance Publique-Hôpitaux de Paris (AP-HP), Unité de Recherche Clinique, Hôpital Cochin, Paris, France; 9 INSERM, UMR_S 1138, Cordeliers Research Center, Université de Paris, Paris, France; 10 Department of Medical Informatics, AP-HP, Centre-Université de Paris, Necker-Enfants Malades Hospital, Paris, France; 11 Division of Epidemiology, Services and Prevention Research, National Institute on Drug Abuse, 6001 Executive Boulevard, Bethesda, MD 20852, USA; 12 Department of Psychiatry, Columbia University/New York State Psychiatric Institute, 1051 Riverside Drive, Unit 69, New York, NY 10032, USA; 13 INSERM, UMS 011, Population-based Epidemiologic Cohorts, Villejuif, France; 14 AP-HP, Hôpital Hôtel-Dieu, DMU Psychiatrie et Addictologie, Service de Psychiatrie de l'adulte, Paris, France; 15 Anesthesia and Intensive Care Department, Hôpitaux Universitaires Paris Île-de-France Ouest, Boulogne-Billancourt, France; 16 Institut de Pharmacologie et de Biologie Structurale, IPBS, Université de Toulouse, Toulouse, France; 17 INSERM U1142 LIMICS, UMRS 1142, Sorbonne Universities, UPMC University of Paris 06, University of Paris 13, Paris, France

**Keywords:** Benzodiazepine, COVID-19, mortality, SARS-CoV-2

## Abstract

**Aims:**

To examine the association between benzodiazepine receptor agonist (BZRA) use and mortality in patients hospitalised for coronavirus disease 2019 (COVID-19).

**Methods:**

A multicentre observational study was performed at Greater Paris University hospitals. The sample involved 14 381 patients hospitalised for COVID-19. A total of 686 (4.8%) inpatients received a BZRA at hospital admission at a mean daily diazepam-equivalent dose of 19.7 mg (standard deviation (s.d.) = 25.4). The study baseline was the date of admission, and the primary endpoint was death. We compared this endpoint between patients who received BZRAs and those who did not in time-to-event analyses adjusted for sociodemographic characteristics, medical comorbidities and other medications. The primary analysis was a Cox regression model with inverse probability weighting (IPW).

**Results:**

Over a mean follow-up of 14.5 days (s.d. = 18.1), the primary endpoint occurred in 186 patients (27.1%) who received BZRAs and in 1134 patients (8.3%) who did not. There was a significant association between BZRA use and increased mortality both in the crude analysis (hazard ratio (HR) = 3.20; 95% confidence interval (CI) = 2.74–3.74; *p* < 0.01) and in the IPW analysis (HR = 1.61; 95% CI = 1.31–1.98, *p* < 0.01), with a significant dose-dependent relationship (HR = 1.55; 95% CI = 1.08–2.22; *p* = 0.02). This association remained significant in sensitivity analyses. Exploratory analyses indicate that most BZRAs may be associated with an increased mortality among patients hospitalised for COVID-19, except for diazepam, which may be associated with a reduced mortality compared with any other BZRA treatment.

**Conclusions:**

BZRA use may be associated with an increased mortality among patients hospitalised for COVID-19, suggesting the potential benefit of decreasing dose or tapering off gradually these medications when possible.

## Introduction

Global spread of the novel coronavirus severe acute respiratory syndrome coronavirus 2 (SARS-CoV-2) has created an unprecedented infectious disease crisis worldwide (Chevance *et al*., 2020; Hoertel *et al*., 2020*a*, 2020*b*, 2021*a*). Benzodiazepine receptor agonists (BZRAs), including benzodiazepines and Z-drugs, potentiate the rapid neuroinhibitory effect of the neurotransmitter gamma-aminobutyric acid in the brain and spinal cord. These medications are commonly prescribed for anxiety and sleep problems and as muscle relaxants and pre-medications in anaesthesia, but are also effective for treating epilepsy, alcohol withdrawal syndrome and acute behavioural disturbance (Mallon *et al*., 2009; Hayhoe and Lee-Davey, 2018). Potential deleterious effects associated with these medications are well documented. Several studies (Belleville, 2010; Obiora *et al*., 2013; Weich *et al*., 2014; Nakafero *et al*., 2015; Palmaro *et al*., 2015; Parsaik *et al*., 2016), although not all (Rumble and Morgan, 1992; Kojima *et al*., 2000; Phillips and Mannino, 2005; Patorno *et al*., 2017), have found a significant association between BZRAs and increased all-cause mortality. BZRAs have also been associated with an increased risk of infection (Joya *et al*., 2009), including pneumonia (Obiora *et al*., 2013), asthma exacerbation (Nakafero *et al*., 2015) and respiratory depression (Roth, 2009). To our knowledge, there are no data on the association of BZRA use with mortality among patients hospitalised for coronavirus disease 2019 (COVID-19). Following a recent release by the US Food and Drug Administration (FDA) of a drug safety communication warning patients and health care providers about the risks of BZRA use (US Food and Drug Administration, 2020), it is important to investigate whether these medications are associated with an increased mortality among patients with COVID-19. If this was the case, a second unanswered question is whether all BZRAs or specific BZRA treatments are associated with this risk. This knowledge might help guide clinicians on the choice of medications for patients with COVID-19 with clinical indications for BZRAs.

In this report, we used data from an observational multicentre retrospective cohort study performed at Greater Paris University hospitals and examined the association between BZRAs and mortality. Our primary hypothesis was that BZRA use would be associated with an increased mortality among patients hospitalised for COVID-19 in time-to-event analyses adjusting for sociodemographic characteristics, medical comorbidities and other medications. Our secondary hypotheses were that this association would be dose-dependent with higher doses conferring greater risk, and at least partially explained by respiratory depression.

## Methods

### Setting and cohort assembly

A multicentre observational retrospective cohort study was conducted at 36 Assistance publique-Hôpitaux de Paris (AP-HP) hospitals (Hoertel *et al*., 2021*c*, 2021*d*, 2021*e*, 2021*f*, 2021*g*, 2021*h*; Sánchez-Rico *et al.*, 2021). We included all adults aged 18 years or over who have been admitted to these medical centres from the beginning of the epidemic in France, i.e. 24th January until 1st May. COVID-19 was ascertained by a positive reverse-transcriptase-polymerase chain reaction (RT-PCR) test from analysis of nasopharyngeal or oropharyngeal swab specimens. This observational study using routinely collected data received approval from the Institutional Review Board of the AP-HP clinical data warehouse (decision CSE-20-20_COVID19, IRB00011591, 8 April 2020) and is part of a broader project aiming at examining the potential associations between psychotropic medications and COVID-19-related mortality, deposited in French at the ‘Entrepôt de Données de Santé’ (EDS) website (https://eds.aphp.fr/recherches-en-cours), which has led to several publications (Hoertel *et al*., 2021*c*, 2021*d*, 2021*e*, 2021*f*, 2021*g*, 2021*h*; Sánchez-Rico *et al.*, 2021). AP-HP clinical Data Warehouse initiatives ensure patient information and informed consent regarding the different approved studies through a transparency portal in accordance with European Regulation on data protection and authorisation no. 1980120 from National Commission for Information Technology and Civil Liberties.

We used data from the AP-HP Health Data Warehouse (‘Entrepôt de Données de Santé (EDS)’). This warehouse contains all available clinical data on all inpatient visits for COVID-19 to any Greater Paris University hospital. The data included patient demographic characteristics, vital signs, laboratory test and RT-PCR test results, medication administration data, medication lists during current and past hospitalisations in AP-HP hospitals, current diagnoses, discharge disposition and death certificates.

### Variables assessed

We obtained the following data for each patient at the time of the hospitalisation through electronic health records (Devlin *et al*., 2019; Jouffroy *et al*., 2020): sex; age, which was categorised into three classes based on age cutoffs (i.e. 18–50, 51–70, 71+) suggested by Williamson *et al*. (2020), who studied factors associated with COVID-19-related mortality using OpenSAFELY, a secure health analytics platform created on behalf of NHS England and covering about 40% of all patients in England; hospital, which was categorised into four classes following the administrative clustering of AP-HP hospitals in Paris and its suburbs based on their geographical location (i.e. AP-HP Centre – Paris University, Henri Mondor University Hospitals and at home hospitalisation; AP-HP Nord and Hôpitaux Universitaires Paris Seine-Saint-Denis; AP-HP Paris Saclay University and AP-HP Sorbonne University); obesity, which was defined as having a body mass index higher than 30 kg/m^2^ or an International Statistical Classification of Diseases and Related Health Problems (ICD-10) diagnosis code for obesity (E66.0, E66.1, E66.2, E66.8, E66.9); self-reported current smoking status; any medical condition associated with increased clinical severity related to COVID-19 and BZRA use (Gordon *et al*., 2020; Hur *et al*., 2020; Ruan *et al*., 2020; Williamson *et al*., 2020), based on ICD-10 diagnosis codes, including diabetes mellitus (E11), diseases of the circulatory system (I00–I99), diseases of the respiratory system (J00–J99), neoplasms (C00–D49), diseases of the blood and blood-forming organs and certain disorders involving the immune mechanism (D5–D8), frontotemporal dementia (G31.0), peptic ulcer (K27), diseases of liver (K70–K95), hemiplegia or paraplegia (G81–G82), acute kidney failure or chronic kidney disease (N17–N19) and HIV (B20); any medication prescribed according to compassionate use or as part of a clinical trial (Haut Conseil de la Santé Publique, 2020) (e.g. hydroxychloroquine, azithromycin, remdesivir, tocilizumab, sarilumab or dexamethasone) and clinical markers of disease severity, including respiratory depression, defined by a respiratory rate <12 breaths/min or a resting peripheral capillary oxygen saturation in ambient air <90%, and any other clinical marker of disease severity, defined as having temperature >40°C or systolic blood pressure <100 mmHg or respiratory rate >24 breaths/min or plasma lactate levels higher than 2 mmol/l (Haut Conseil de la Santé Publique, 2020). For these last two variables, a third category for missing data was added. Additionally, to take into account possible confounding by indication bias for BZRAs, we recorded whether patients had any current psychiatric disorder (F00–F99) based on ICD-10 diagnosis codes, and whether they were prescribed any other psychotropic medication, including any antidepressant (Hoertel *et al*., 2021*d*), mood stabiliser (i.e. lithium or antiepileptic medications with mood stabilising effects) or antipsychotic medication (Hoertel *et al*., 2021*f*, 2021*g*).

All medical notes and prescriptions are computerised in Greater Paris University hospitals. Medications including their dosage, frequency, date and mode of administration were identified from medication administration data or scanned hand-written medical prescriptions, through two deep learning models based on bidirectional encoder representations from transformer (BERT) contextual embeddings (Devlin *et al*., 2019), one for the medications and another for their mode of administration. Contextualised word embeddings from BERT allow to project dense vector representations of words in lower dimensional spaces while maintaining semantic and contextual importance of a word in a numeric form (Devlin *et al*., 2019). This allows us to automatise the data collection, while significantly increasing its reliability (Devlin *et al*., 2019). The model was trained on the APmed corpus (Jouffroy *et al*., 2020), a previously annotated dataset for this task. Extracted medication names were then normalised to the anatomical therapeutic chemical terminology using approximate string matching.

### BZRA use

Study baseline was defined as the date of hospital admission. To minimise the risk of immortal time bias, BZRA use was defined as receiving these medications at baseline. To be considered at baseline, BZRA use had to meet two conditions: (i) a first prescription of BZRA from baseline to up to 48 h from hospital admission and (ii) strictly before (in min) the exact moment of the end of the index hospitalisation or death. Patients with a BZRA prescription that did not meet these two conditions were excluded from the analysis. We chose this first condition to reduce the risk of immortal time bias that could result from the inclusion of patients who were first prescribed this medication lately and thus being by definition alive at that time, and we used 48 h delay because we considered that, in a context of overwhelmed hospital units during the COVID-19 peak incidence, all patients may not have received or been prescribed their usual medication regimens the first day of their hospital admission, or this treatment may not have been recorded at this time. We chose the second condition to ensure that BZRA exposure precedes the outcome or the end of the index hospitalisation.

### Primary endpoint

The primary endpoint was the time from study baseline to death. Patients without an end-point event had their data censored on 1st May 2020.

### Statistical analysis

We calculated frequencies of all baseline characteristics described above in patients receiving or not receiving BZRAs and compared them using standardised mean differences (SMDs). We considered SMDs >0.1 as reflecting substantial differences, a recommended threshold for declaring imbalance (Austin, 2009).

To examine the association between BZRA use and the endpoint of death, we performed Cox proportional-hazards regression models (Therneau and Grambsch, 2000). To help account for the non-randomised prescription of BZRAs and reduce the effects of confounding, the primary analysis used propensity score analysis with inverse probability weighting (IPW) (Robins *et al*., 2000; Geleris *et al*., 2020). The individual propensities for receiving any BZRA were estimated using a multivariable logistic regression model that included patient characteristics and other medications. In the IPW analyses, the predicted probabilities from the propensity-score models were used to calculate the stabilised IPW (Geleris *et al*., 2020). In the main analysis, the association between BZRA use and the endpoint was then estimated using an IPW Cox regression model. In the case of non-balanced covariates, an IPW multivariable Cox regression model adjusting for the non-balanced covariates was also performed. Kaplan–Meier curves were obtained using the IPW (Efron, 1981; Kassambara *et al*., 2020), and their pointwise 95% confidence intervals (CIs) were estimated using the non-parametric bootstrap method (Kassambara *et al*., 2020).

We conducted five sensitivity analyses to examine the robustness of the results from the main analysis. First, we performed a multivariable Cox regression model including as covariates the same variables as in the IPW analysis. Second, we used a univariate Cox regression model in a matched analytic sample using a 1 : 1 ratio, based on the same variables used for the IPW analysis and the multivariable Cox regression analysis. To reduce the effects of confounding, optimal matching was used to obtain the smallest average absolute distance across all clinical characteristics between the exposed patients and non-exposed matched controls. Third, to examine a potential indication bias of prescription of BZRAs in intensive care units (ICUs) as a possible treatment for palliative care or as an aid to oral intubation, we reproduced the main analyses after excluding all patients who had been hospitalised in ICUs. Fourth, we examined whether our findings were similar to models imputing missing data using multiple imputation (Stekhoven and Buehlmann, 2012) instead of excluding patients with any missing data as in the main analyses. Finally, to examine the potential influence of excluding patients who received a BZRA after 48 h from hospital admission while still accounting for a potential immortal time bias, we reproduced the main analysis while including all participants who received a BZRA at any time, and classifying BZRA use as a time-dependent variable (Therneau and Grambsch, 2000; Dekker *et al*., 2008). This analysis allows comparisons of the risk of occurrence of mortality between groups at each event time by re-evaluating study group based on whether participants had first received a BZRA by that time. Thus, patients enter the exposed group at the time of actual first initiation of the treatment. For example, a participant who was first prescribed a BZRA at 72 h from hospital admission is considered as non-exposed from baseline until 72 h, and as exposed from 72 h until the end of the study. For all analyses, a weighted Cox regression model was used when proportional hazards assumption was not met (Dunkler *et al*., 2018).

Finally, we performed additional exploratory analyses. First, we searched for a potential dose-dependent relationship by testing the association between the daily dose of BZRA received during the first day of prescription (converted into diazepam-equivalent dose (Ashton, 2002) and dichotomised at the mean value) and the endpoint, adjusted for the same covariates used in the main analysis, among patients who received a BZRA at baseline. Second, we examined whether respiratory depression or other clinical markers of disease severity may at least partly explain this association by adjusting successively for these variables. Finally, we examined the relationships between each BZRA and mortality using the same statistical approach as described for the main analysis.

For all associations, we performed residual analyses to assess the fit of the data, checked assumptions, including proportional hazards assumption using proportional hazards tests and diagnostics based on weighted residuals (Grambsch and Therneau, 1994; Therneau and Grambsch, 2000), and examined the potential influence of outliers. To improve the quality of result reporting, we followed the recommendations of The Strengthening the Reporting of Observational Studies in Epidemiology Initiative. Because our main hypothesis focused on the association between BZRA use and mortality, and was tested in a single model in the main analysis, statistical significance was fixed at two-sided *p*-value <0.05. As described above, we planned to perform additional exploratory analyses only if a significant association was found in the main analysis. All analyses were conducted in R software version 2.4.3 (R Project for Statistical Computing).

## Results

### Characteristics of the cohort

Of the 17 131 patients with a positive COVID-19 RT-PCR test who had been hospitalised for COVID-19, 1963 patients (11.5%) were excluded because of missing data or their young age (i.e. <18 years old). In addition, of the 1473 patients who received a BZRA at any time during the visit, 787 were excluded because they were prescribed it more than 48 h after hospital admission. Of the remaining 14 381 adult patients, 686 (4.8%) received a BZRA in the first 48 h of hospitalisation at a mean diazepam-equivalent dose of 19.7 mg per day (standard deviation (s.d.) = 25.4, median = 10.0, lower quartile = 5.0, higher quartile = 22.9, range = 1.85–240.0 mg) (online Supplementary Fig. S1). Of these 686 patients, 41.1% (*N* = 282) had a prescription of BZRA during a prior hospitalisation at AP-HP in the past 6 months, and 36.4% (*N* = 183) received at least two BZRA medications. The median delay from hospital admission to first prescription of BZRA was 0.94 days (s.d. = 0.55).

Over a mean follow-up of 14.5 days (median = 7 days; interquartile range (IQR) = 24; s.d. = 18.1), 1320 patients (9.2%) had an end-point event at the time of data cutoff on 1st May. Among patients who received a BZRA, the mean follow-up was 12.9 days (IQR = 13; s.d. = 13.1; median = 8 days), while it was of 14.5 days (IQR = 24; s.d. = 18.3; median = 7 days) in those who did not.

Associations between baseline characteristics and the endpoint are shown in online Supplementary Table S1. The distributions of patient characteristics according to BZRA use are shown in Table 1. In the full sample, BZRA use significantly differed according to all characteristics except for sex ratio, and the direction of the associations indicated older age and greater medical severity of patients receiving any BZRA. After applying the propensity score weights, these differences were substantially reduced. In the matched analytic sample, there were no significant differences in any characteristic (Table 1).

**Table 1.** Characteristics of adult patients hospitalised for COVID-19 receiving or not receiving a BZRA at hospital admission (*N* =  14 381)

	Exposed to any BZRA (*N* = 686)	Not exposed to any BZRA (*N* = 13 695)	Non-exposed matched group (*N* = 686)	Exposed to any BZRA *v*. not exposed	Exposed to any BZRA *v*. not exposed	Exposed to any BZRA *v*. non-exposed matched group
				Crude analysis	Analysis weighted by IPW	Matched analytic sample analysis
	*N* (%)	*N* (%)	*N* (%)	SMD	SMD	SMD
Age				0.893	0.174	0.029
18–50 years	70 (10.2%)	5669 (41.4%)	65 (9.4%)			
51–70 years	192 (28.0%)	4395 (32.1%)	198 (28.9%)			
More than 70 years	424 (61.8%)	3631 (26.5%)	423 (61.7%)			
Sex				0.047	0.095	0.108
Women	348 (50.7%)	7270 (53.1%)	311 (45.3%)			
Men	338 (49.3%)	6425 (46.9%)	375 (54.7%)			
Hospital				0.387	0.050	0.063
AP-HP Centre – Paris University, Henri Mondor University Hospitals and at home hospitalisation	206 (30.0%)	6585 (48.1%)	212 (30.9%)			
AP-HP Nord and Hôpitaux Universitaires Paris Seine-Saint-Denis	222 (32.4%)	3685 (26.9%)	227 (33.1%)			
AP-HP Paris Saclay University	122 (17.8%)	1575 (11.5%)	106 (15.5%)			
AP-HP Sorbonne University	136 (19.8%)	1850 (13.5%)	141 (20.6%)			
Obesity^a^				0.186	0.074	0.079
Yes	135 (19.7%)	1758 (12.8%)	114 (16.6%)			
No	551 (80.3%)	11 937 (87.2%)	572 (83.4%)			
Smoking^b^				0.263	0.074	0.065
Yes	112 (16.3%)	1072 (7.83%)	96 (14.0%)			
No	574 (83.7%)	12 623 (92.2%)	590 (86.0%)			
Any medical condition^c^				0.711	0.058	0.027
Yes	393 (57.3%)	3336 (24.4%)	402 (58.6%)			
No	293 (42.7%)	10 359 (75.6%)	284 (41.4%)			
Medication according to compassionate use or as part of a clinical trial^d^				0.371	0.103	0.017
Yes	170 (24.8%)	1483 (10.8%)	165 (24.1%)			
No	516 (75.2%)	12 212 (89.2%)	521 (75.9%)			
Any current psychiatric disorder^e^				0.582	0.052	0.007
Yes	154 (22.4%)	498 (3.6%)	152 (22.2%)			
No	532 (77.6%)	13 197 (96.4%)	534 (77.8%)			
Any antidepressant				1.045	0.185	0.075
Yes	285 (41.5%)	411 (3.0%)	260 (37.9%)			
No	401 (58.5%)	13 284 (97.0%)	426 (62.1%)			
Any mood stabiliser medication^f^				0.617	0.404	0.011
Yes	143 (20.8%)	284 (2.1%)	140 (20.4%)			
No	543 (79.2%)	13 411 (97.9%)	546 (79.6%)			
Any antipsychotic medication				0.714	0.305	0.014
Yes	163 (23.8%)	198 (1.4%)	159 (23.2%)			
No	523 (76.2%)	13 497 (98.6%)	527 (76.8%)			

SMD, standardised mean difference.

a Defined as having a body-mass index higher than 30 kg/m^2^ or an ICD-10 diagnosis code for obesity (E66.0, E66.1, E66.2, E66.8, E66.9).

b Current smoking status was self-reported.

c Assessed using ICD-10 diagnosis codes for diabetes mellitus (E11), diseases of the circulatory system (I00–I99), diseases of the respiratory system (J00–J99), neoplasms (C00-D49), diseases of the blood and blood-forming organs and certain disorders involving the immune mechanism (D5–D8), frontotemporal dementia (G31.0), peptic ulcer (K27), diseases of liver (K70–K95), hemiplegia or paraplegia (G81–G82), acute kidney failure or chronic kidney disease (N17–N19) and HIV (B20).

d Any medication prescribed as part of a clinical trial or according to compassionate use (e.g. hydroxychloroquine, azithromycin, remdesivir, tocilizumab, sarilumab or dexamethasone).

e Assessed using ICD-10 diagnosis codes (F00–F99).

f Included lithium and antiepileptic medications with mood stabilising properties.

SMD > 0.1 indicates substantial difference.

### Study endpoint

**Fig. 1.**
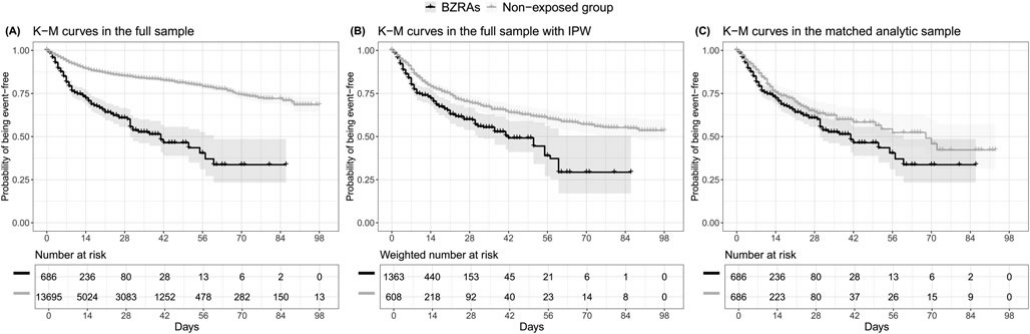
Kaplan–Meier curves for mortality in the full sample crude analysis (*N* = 14 381) (A), in the full sample analysis with IPW (*N* = 14 381) (B), and in the matched analytic sample using a 1 : 1 ratio (*N* = 1372) (C), according to BZRA use at baseline, among adult patients hospitalised for COVID-19. The shaded areas represent pointwise 95% CIs. Numbers at risk in panel B are weighted.

The endpoint of death occurred in 186 patients (27.1%) who received a BZRA at baseline and 1134 patients (8.3%) who did not. The crude, unadjusted analysis (hazard ratio (HR) = 3.20; 95% CI = 2.74–3.74; *p* < 0.001), the primary analysis with IPW (HR = 1.61; 95% CI = 1.31–1.98; *p* < 0.001) and the multivariable IPW Cox regression adjusting for unbalanced covariates (HR = 1.56; 95% CI = 1.29–1.89; *p* < 0.001) showed a significant association between BZRA use and increased mortality (Fig. 1; Table 2).

**Table 2.** Association between BZRA use at baseline and mortality among adult patients hospitalised for COVID-19

	Number of events/number of patients	Crude Cox regression analysis	Multivariable Cox regression analysis	Analysis weighted by IPW	Analysis weighted by IPW adjusted for unbalanced covariates^a^	Number of events/number of patients in the matched groups	Univariate Cox regression in a 1 : 1 ratio matched analytic sample	Cox regression in a 1 : 1 ratio matched analytic sample adjusted for unbalanced covariates^b^
	*N*/*N* (%)	HR (95% CI; *p*-value)	HR (95% CI; *p*-value)	HR (95% CI; *p*-value)	HR (95% CI; *p*-value)	*N*/*N* (%)	HR (95% CI; *p*-value)	HR (95% CI; *p*-value)
No BZRA	1134/13 695 (8.3%)	Ref.	Ref.	Ref.	Ref.	143/686 (20.8%)	Ref.	Ref.
Any BZRA	186/686 (27.1%)	3.20 (2.74–3.74; <0.001*)	1.94 (1.45–2.59; <0.001*)	1.61 (1.31–1.98; <0.001*)	1.56 (1.29–1.89; <0.001*)	186/686 (27.1%)	1.34 (1.08–1.67; 0.009*)	1.36 (1.09–1.69; 0.006*)

BZRA, benzodiazepine receptor agonist; HR, hazard ratio; CI, confidence interval.

*Two-sided *p*-value is significant (*p* < 0.05).

a Adjusted for age, medication according to compassionate use or as part of a clinical trial, any mood stabiliser medication and any antipsychotic medication.

b Adjusted for sex.

In sensitivity analyses, the multivariable Cox regression model in the full sample also indicated a significant association (HR = 1.94; 95% CI = 1.45–2.59; *p* < 0.001), as did the univariate Cox regression model in a matched analytic sample using a 1 : 1 ratio (HR = 1.34; 95% CI = 1.08–1.67; *p* = 0.009) (Table 2). Similarly, the primary analysis using imputed data yielded significant results (online Supplementary Table S2), as did that considering BZRA use as a time-dependent variable and including all patients who received a BZRA at any time during the visit (online Supplementary Table S3). The exclusion from the analyses of the patients who had been admitted to ICUs did not alter the significance of the association (online Supplementary Table S4).

Additional analyses showed a significant dose-dependent relationship between baseline daily BZRA dose and the endpoint (HR = 1.55; 95% CI = 1.08–2.22; *p* = 0.017), based on the primary IPW analysis. Additional adjustments for respiratory depression, any other clinical markers of disease severity, or both, resulted in still significant associations, which were of similar magnitude to that observed in the primary analysis (online Supplementary Table S5). We found that all individual BZRAs were significantly associated with an increased mortality, except for diazepam (online Supplementary Table S6). Following adjustments, there were significant associations of any BZRA other than diazepam and midazolam with an increased mortality, as compared to not receiving BZRAs (online Supplementary Table S6). Finally, compared with any other BZRA treatment, diazepam use was significantly associated with a reduced mortality in the crude analysis (HR = 0.44; 95% CI = 0.23–0.86; *p* = 0.017), in the primary analysis (HR = 0.31; 95% CI = 0.13–0.74; *p* = 0.008), and in the sensitivity analyses (online Supplementary Fig. S2; Tables S6–S8).

## Discussion

In this multicentre retrospective observational study involving a large sample of patients hospitalised for COVID-19, we found that BZRA use was significantly and substantially associated with an increased mortality, independently of patient characteristics and other medications, and with a significant dose-dependent relationship. This association remained significant in multiple sensitivity analyses. Exploratory analyses suggested that most BZRAs could be associated with this risk in these patients, except for diazepam, which may be associated with a reduced mortality compared with any other BZRA treatment.

We found that BZRA use was significantly associated with an increased mortality among patients hospitalised for COVID-19. This association might be explained by several mechanisms. First, although the risk of respiratory depression associated with benzodiazepines in the general population is debated and was not found to play a substantial role in our study, it might be relevant among elderly patients with COVID-19 and in those with pre-existing comorbidities, such as chronic obstructive pulmonary disease (Ostuzzi *et al*., 2020). Second, benzodiazepines may be associated with an increased risk of secondary infections, and particularly pneumonia, in patients with COVID-19 (Sun *et al*., 2019). Finally, in patients with COVID-19 and known risk factors for delirium (e.g. old age, dementia and multiple comorbidities), BZRA use may favour the occurrence of this condition (Ostuzzi *et al*., 2020), which is associated with unfavourable COVID-19 disease prognosis (Chen *et al*., 2020). These results suggest the need to carefully reevaluate the indication of BZRAs and decrease in dose or taper these medications when possible in patients with COVID-19 (Hayhoe and Lee-Davey, 2018).

Exploratory analyses indicate that most BZRAs may be associated with an increased mortality among patients hospitalised for COVID-19, except for diazepam. This exception could be in line with preclinical findings. Prior studies have indicated a central role of acid sphingomyelinase/ceramide system in SARS-CoV-2 infections (Carpinteiro *et al*., 2020, 2021; Hoertel *et al*., 2021*b*; Kornhuber *et al*., 2021) and that the formation of ceramide-enriched membrane platforms mediates the entry of the virus into epithelial cells (Kornhuber *et al*., 2021). Furthermore, prior work indicated that plasma levels of ceramides are significantly and substantially associated with COVID-19 clinical severity and inflammation markers in patients with COVID-19 (Marín-Corral *et al*., 2021; Torretta *et al*., 2021). Finally, prior observational studies reported that taking a FIASMA medication upon hospital admission is associated with a reduced likelihood of intubation or death (Darquennes *et al*., 2021; Hoertel *et al*., 2021*c*, 2021*h*). Although most BZRAs do not influence cellular ceramide levels, diazepam has been shown to interact with the acyl coenzyme A binding protein, also known as ‘diazepam binding inhibitor’, a protein that contributes to ceramide synthesis (Guidotti *et al*., 1983; Ferreira *et al*., 2017). This interaction may result in a reduced activity of the ceramide synthase pathway and, finally, reduced cellular ceramide concentration, which might possibly explain the reduced adverse effects of diazepam on COVID-19 compared to other BZRAs. However, this result should be interpreted with caution and other studies are required to confirm this finding and this potential underlying mechanism.

Our study has several limitations. First, there are two possible major inherent biases in observational studies: unmeasured confounding and confounding by indication. We tried to minimise the effects of confounding in several different ways. First, we used an analysis with IPW to minimise the effects of confounding by indication (Robins *et al*., 2000; Geleris *et al*., 2020). Second, we performed multiple sensitivity analyses, which showed similar results. Finally, although some amount of unmeasured confounding may remain, our analyses adjusted for numerous potential confounders. Other limitations include missing data for some baseline characteristic variables (i.e. 11.5%), which might be explained by the overwhelming of all hospital units during the COVID-19 peak incidence, and different results might have been observed during a lower COVID-19 incidence period. However, imputation of missing data did not alter the significance of our results. Second, BZRA use was defined as receiving these medications within the first 48 h from hospital admission and before the end of the index hospitalisation or death. We used this delay because we considered that, in a context of overwhelmed hospital units during the COVID-19 peak incidence, all patients may not have received or been prescribed their usual medication regimens the first day of their hospital admission, or this treatment may not have been recorded at this time. This delay may have introduced an immortal time bias. However, this bias is likely to have biased the results towards the null hypothesis, leading to potentially underestimate the strength of the association, and similar results were found when considering BZRA use as a time-dependent variable. Third, although this study is part of a broader project to examine potential associations between the use of psychotropic medications and COVID-19-related mortality, deposited at the EDS website (https://eds.aphp.fr/recherches-en-cours), the specific protocol of the present study has not been deposited in a public domain. Therefore, no comparison can be done between the current analyses with a pre-specified protocol, and inflation of type I error might have occurred due to multiple testing across psychotropic medication classes and molecules. However, in these exploratory analyses, we are primarily interested in results to generate new hypotheses. In this context, it may be more important to explore leads that might ultimately turn out to be incorrect rather than miss potentially important findings (Rothman, 2014). Also, we performed several sensitivity analyses that yielded similar results. Fourth, inflation of type I error might have occurred in secondary analyses due to multiple testing. Fifth, our study cannot establish a causal relationship between BZRA use and increased mortality (Le Strat and Hoertel, 2011). Finally, despite the multicentre design, our results may not be generalisable to outpatients or other regions.

Our findings indicate that BZRA use may be associated with an increased mortality among patients hospitalised for COVID-19 and suggest a potential benefit of decreasing dose or tapering off gradually these medications when possible in these patients.

## Supplementary material

For supplementary material accompanying this paper visit https://doi.org/10.1017/S2045796021000743. click here to view supplementary material

## Availability of data and materials

Data from the AP-HP Health Data Warehouse can be obtained upon request at https://eds.aphp.fr//.
