# Supplementary material for: Association between benzodiazepine receptor agonist use and mortality in patients hospitalised for COVID-19: a multicentre observational study
Source: Epidemiol Psychiatr Sci. 2022 Mar 30;31:e18. doi: 10.1017/S2045796021000743 (PMC8967698; doi:10.1017/S2045796021000743)
Supplement: Supplementary file 1 [file S2045796021000743sup001.docx]

**SUPLEMENTARY MATERIAL**

[Figure S1. Study cohort. 2](#_Toc71275921)

[Figure S2. Kaplan-Meier curves for mortality in the full sample crude analysis (N=686) (A), in the full sample analysis with inverse probability weighting (N=686) (B) and in the matched analytic sample using a 1:1 ratio (N=148) (C) of patients who had been hospitalized for COVID-19, according to diazepam or other benzodiazepine receptor agonist (BZRA) use at baseline. 3](#_Toc71275922)

[Table S1. Associations of baseline characteristics with the endpoint of death in the cohort of adult patients who had been hospitalized for COVID-19 (N=14,381). 4](#_Toc71275923)

[Table S2. Association between benzodiazepine receptor agonist (BZRA) use at baseline and mortality in models imputing missing data using multiple imputation. 7](#_Toc71275924)

[Table S3. Associations between BZRA use and mortality in patients hospitalized for COVID-19, when including all patients who received a BZRA and considering BZRA use as a time-dependent variable. 8](#_Toc71275925)

[Table S4. Association between benzodiazepine receptor agonist (BZRA) use and the endpoint of death among patients who had been hospitalized for COVID-19 outside ICUs (N = 13,693). 9](#_Toc71275926)

[Table S5. Association between benzodiazepine receptor agonist (BZRA) use at baesline and mortality, following additional adjustments for respiratory depression, any other clinical markers of disease severity, or both. 10](#_Toc71275927)

[Table S7. Characteristics of patients with COVID-19 receiving diazepam versus any other benzodiazepine receptor agonist (BZRA) (N=686). 13](#_Toc71275928)

[Table S8. Association between diazepam use and the endpoint of death among patients who had been hospitalized for COVID-19 and had received benzodiazepine receptor agonists at baseline (N=686). 16](#_Toc71275929)

# Figure S1. Study cohort.

17,131 patients with a positive COVID-19 RT-PCR test

who had been hospitalized for COVID-19 from January 24^th^ to May 1^st^

1,963 patients were excluded because of missing data or age:

- Hospitalization dates: N = 457

- Smoking status: N = 1,319

- Sex: N = 5

- Aged less than 18 years: N = 212

-

15,168 adult inpatients (1,473 received a benzodiazepine receptor agonist (BZRA) treatment and 13,695 did not)

787 patients who received a BZRA during the visit were excluded because the treatment started after study baseline (i.e. 48 hours after hospital admission) (N = 559) or information on prescription (e.g., date of prescription, dose) was missing (N = 228)

Benzodiazepine receptor agonist at baseline

(N = 686)

14,381 adult inpatients included in the propensity and regression analyses

No benzodiazepine receptor agonist during the visit (N = 13,695)

Oxazepam (N = 234)

Alprazolam (N = 129)

Midazolam (N = 125)

Zopiclone (N = 175)

Diazepam (N = 74)

Clonazepam (N=43)

Prazepam (N = 25)

Clobazam (N = 23)

Zolpidem (N = 23)

Lormetazepam (N = 21)

Bromazepam (N = 19)

Lorazepam (N=17)

Clorazepate (N = 12)

Clotiazepam (N = 1)

Loprazolam (N = 1)

Nitrazepam (N = 1)

Nordazepam (N = 1)

# Figure S2. Kaplan-Meier curves for mortality in the full sample crude analysis (N=686) (A), in the full sample analysis with inverse probability weighting (N=686) (B) and in the matched analytic sample using a 1:1 ratio (N=148) (C) of patients who had been hospitalized for COVID-19, according to diazepam or other benzodiazepine receptor agonist (BZRA) use at baseline.


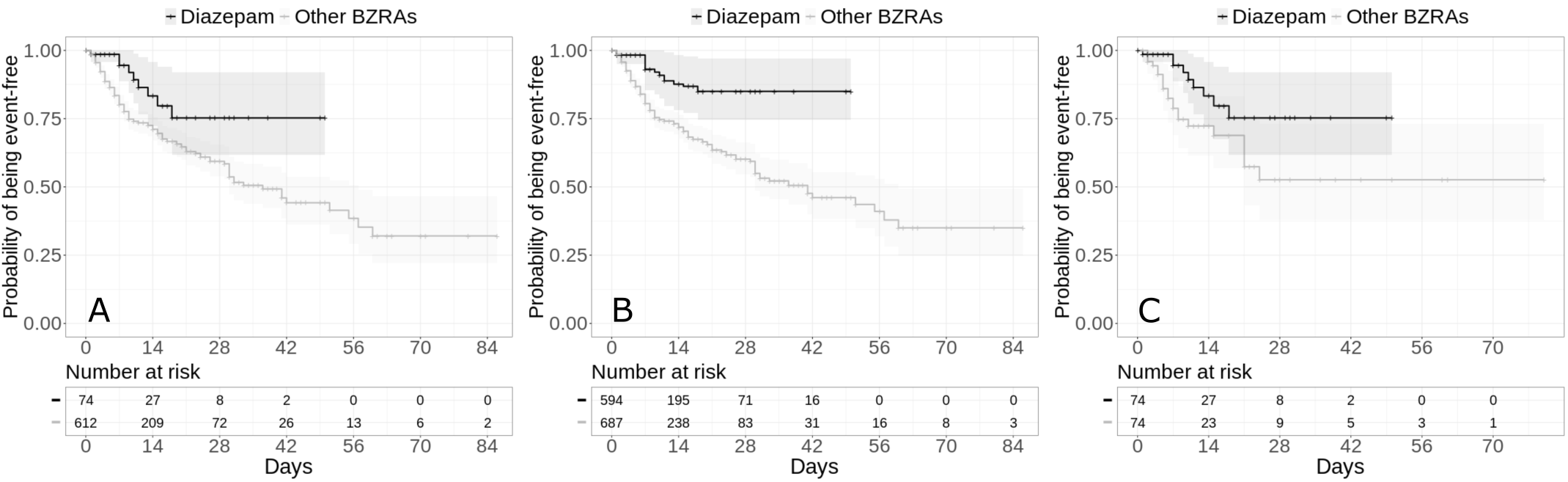


The shaded areas represent pointwise 95% confidence intervals. Numbers at risk in the panel B are weighted.

# Table S1. Associations of baseline characteristics with the endpoint of death in the cohort of adult patients who had been hospitalized for COVID-19 (N=14,381).

|  |  |  |  | **Endpoint of death** | | |
| --- | --- | --- | --- | --- | --- | --- |
|  | **Full population**  **(N =14,381)** | **With the end-point event**  **(N =1,320)** | **Without the end-point event**  **(N =13,061)** | **Crude analysis** | **Multivariable analysis ^µ^** | |
|  | **N (%)** | **N (%)** | **N (%)** | **HR (95% CI; p-value)** | **HR (95% CI; p-value)** | **Collinearity diagnostics (variance inflation factor)** |
| Age |  |  |  |  |  | 1.02 |
| *18 to 50 years* | 5739 (39.9%) | 47 (3.56%) | 5692 (43.6%) | Ref. | Ref. |  |
| *51 to 70 years* | 4587 (31.9%) | 322 (24.4%) | 4265 (32.7%) | 8.23 (6.06 - 11.18; <0.001*) | 3,14 (1,46 - 6,76; 0,003*) |  |
| *More than 70 years* | 4055 (28.2%) | 951 (72.0%) | 3104 (23.8%) | 24.79 (18.49 - 33.25; <0.001*) | 7,44 (3,70 - 14,97; <0,001*) |  |
| Sex |  |  |  |  |  | 1.05 |
| *Women* | 7618 (53.0%) | 484 (36.7%) | 7134 (54.6%) | Ref. | Ref. |  |
| *Men* | 6763 (47.0%) | 836 (63.3%) | 5927 (45.4%) | 2.02 (1.81 - 2.26; <0.001*) | 1,72 (1,31 - 2,24; <0,001*) |  |
| Hospital |  |  |  |  |  | 1.03 |
| *AP-HP Centre – Paris University, Henri Mondor University Hospitals and at home hospitalization* | 6791 (47.2%) | 412 (31.2%) | 6379 (48.8%) | Ref. | Ref. |  |
| *AP-HP Nord and Hôpitaux Universitaires Paris Seine-Saint-Denis* | 3907 (27.2%) | 450 (34.1%) | 3457 (26.5%) | 2.28 (1.99 - 2.6; <0.001*) | 1,69 (1,25 - 2,28; 0,001*) |  |
| *AP-HP Paris Saclay University* | 1697 (11.8%) | 231 (17.5%) | 1466 (11.2%) | 2.24 (1.91 - 2.64; <0.001*) | 1,06 (0,70 - 1,63; 0,771) |  |
| *AP-HP Sorbonne University* | 1986 (13.8%) | 227 (17.2%) | 1759 (13.5%) | 2.01 (1.71 - 2.37; <0.001*) | 0,96 (0,70 - 1,33; 0,821) |  |
| Obesity ^α^ |  |  |  |  |  | 1.03 |
| *Yes* | 1893 (13.2%) | 279 (21.1%) | 1614 (12.4%) | 1.54 (1.35 - 1.76; <0.001*) | 1,04 (0,77 - 1,40 - 0,818) |  |
| *No* | 12488 (86.8%) | 1041 (78.9%) | 11447 (87.6%) | Ref. | Ref. |  |
| Smoking ^β^ |  |  |  |  |  | 1.02 |
| *Yes* | 1184 (8.2%) | 199 (15.1%) | 985 (7.54%) | 1.73 (1.49 - 2.01; <0.001*) | 0,80 (0,54 - 1,18; 0,259) |  |
| *No* | 13197 (91.8%) | 1121 (84.9%) | 12076 (92.5%) | Ref. | Ref. |  |
| Any medical condition ^γ^ |  |  |  |  |  | 1.20 |
| *Yes* | 3729 (25.9%) | 903 (68.4%) | 2826 (21.6%) | 7.25 (6.43 - 8.16; <0.001*) | 3,17 (2,36 - 4,27; <0,001*) |  |
| *No* | 10652 (74.1%) | 417 (31.6%) | 10235 (78.4%) | Ref. | Ref. |  |
| Medication according to compassionate use or as part of a clinical trial ^ϴ^ |  |  |  |  |  | 1.04 |
| *Yes* | 1653 (11.5%) | 277 (21.0%) | 1376 (10.5%) | 1.94 (1.7 - 2.22; <0.001*) | 0,92 (0,74 - 1,15; 0,476) |  |
| *No* | 12728 (88.5%) | 1043 (79.0%) | 11685 (89.5%) | Ref. | Ref. |  |
| Any current psychiatric disorder ^¥^ |  |  |  |  |  | 1.13 |
| *Yes* | 652 (4.5%) | 240 (18.2%) | 412 (3.15%) | 4.86 (4.22 - 5.6; <0.001*) | 1,57 (1,20 - 2,06; 0,001*) |  |
| *No* | 13729 (95.5%) | 1080 (81.8%) | 12649 (96.8%) | Ref. | Ref. |  |
| Any antidepressant |  |  |  |  |  | 1.07 |
| *Yes* | 696 (4.8%) | 155 (11.7%) | 541 (4.1%) | 2.27 (1.92 - 2.69; <0.001*) | 1,05 (0,76 - 1,44; 0,783) |  |
| *No* | 13685 (95.2%) | 1165 (88.3%) | 12520 (95.9%) | Ref. | Ref. |  |
| Any mood stabilizer medication ^Ω^ |  |  |  |  |  | 1.03 |
| *Yes* | 427 (2.97%) | 77 (5.8%) | 350 (2.7%) | 1.74 (1.38 - 2.19; <0.001) | 0,91 (0,58 - 1,41; 0,660) |  |
| *No* | 13954 (97.0%) | 1243 (94.2%) | 12711 (97.3%) | Ref. | Ref. |  |
| Any antipsychotic medication |  |  |  |  |  | 1.04 |
| *Yes* | 361 (2.5%) | 65 (4.9%) | 296 (2.3%) | 1.77 (1.38 - 2.27; <0.001*) | 0,73 (0,44 - 1,21; 0,225) |  |
| *No* | 14020 (97.5%) | 1255 (95.1%) | 12765 (97.7%) | Ref. | Ref. |  |
| Respiratory depression ^£^ |  |  |  |  |  | 1.45 |
| *Yes* | 387 (2.69%) | 153 (11.6%) | 234 (1.79%) | 2.5 (2.09 - 2.99; <0.001*) | 1,49 (1,07 - 2,06; 0,017*) |  |
| *No* | 3560 (24.8%) | 547 (41.4%) | 3013 (23.1%) | Ref. | Ref. |  |
| *Missing* | 10434 (72.6%) | 620 (47.0%) | 9814 (75.1%) | 0.32 (0.28 - 0.36; <0.001*) | 1,16 (0,87 - 1,55; 0,311) |  |
| Any other clinical marker of severity ^§^ |  |  |  |  |  | 1.45 |
| *Yes* | 2079 (14.5%) | 526 (39.8%) | 1553 (11.9%) | 2.13 (1.84 - 2.47; <0.001*) | 2,08 (1,59 - 2,71; <0,001*) |  |
| *No* | 2282 (15.9%) | 263 (19.9%) | 2019 (15.5%) | Ref. | Ref. |  |
| *Missing* | 10020 (69.7%) | 531 (40.2%) | 9489 (72.7%) | 0.38 (0.33 - 0.44; <0.001*) | 0,83 (0,59 - 1,17; 0,284) |  |

^α^ Defined as having a body-mass index higher than 30 kg/m^2^ or an International Statistical Classification of Diseases and Related Health Problems (ICD-10) diagnosis code for obesity (E66.0, E66.1, E66.2, E66.8, E66.9).

^β^ Current Smoking status was self-reported.

^γ^ Assessed using ICD-10 diagnosis codes for diabetes mellitus (E11), diseases of the circulatory system (I00-I99), diseases of the respiratory system (J00-J99), neoplasms (C00-D49), diseases of the blood and blood-forming organs and certain disorders involving the immune mechanism (D5-D8), frontotemporal dementia (G31.0), peptic ulcer (K27), diseases of liver (K70-K95), hemiplegia or paraplegia (G81-G82), acute kidney failure or chronic kidney disease (N17-N19), and HIV (B20).

^ϴ^ Any medication prescribed as part of a clinical trial or according to compassionate use (e.g., hydroxychloroquine, azithromycin, remdesivir, tocilizumab, sarilumab or dexamethasone).

^¥^ Assessed using ICD-10 diagnosis codes (F00-F99).

^Ω^ Included lithium and antiepileptic medications with mood stabilizing properties.

^µ^ Adjusted for sex, age, hospital type, obesity, current smoking status, any significant medical or psychiatric condition, any medication prescribed according to compassionate use or as part of a clinical trial and other psychotropic medications (i.e. any antidepressant, mood stabilizer and antipsychotic medication).

^£^ Defined by a respiratory rate < 12 breaths/min or a resting peripheral capillary oxygen saturation in ambient air < 90%,

^§^ Defined by a temperature > 40°C or a systolic blood pressure < 100 mmHg or a respiratory rate > 24 breaths/min or a plasma lactate levels higher than 2 mmol/L

* two-sided p-value is significant (p<0.05).

Abbreviations: HR, hazard ratio; SE, standard error; VIF, variance inflation factor.

# Table S2. Association between benzodiazepine receptor agonist (BZRA) use at baseline and mortality in models imputing missing data using multiple imputation.

|  | **Number of events / Number of patients** | **Crude Cox**  **regression analysis** | **Multivariable Cox regression analysis** | **Analysis weighted by inverse-probability-weighting weights** | **Analysis weighted by inverse-probability-weighting weights adjusted for unbalanced covariates ^a^** | **Number of events /**  **Number of patients in the matched groups** | **Univariate Cox regression in a 1:1 ratio matched analytic sample** |
| --- | --- | --- | --- | --- | --- | --- | --- |
|  | N (%) | HR (95% CI; p-value) | HR (95% CI; p-value) | HR (95% CI; p-value) | HR (95% CI; p-value) | N (%) | HR (95% CI; p-value) |
| No BZRA | 1,299 / 15,602 (8.3%) | Ref. | Ref. | Ref. | Ref. | 148 / 705 (21.0%) | Ref. |
| Any BZRA | 190 / 705 (27.0%) | 3.15 (2.71 - 3.67; <0.001*) | 1.82 (1.33 – 2.48; <0.001*) | 1.51 (1.23 – 1.84; <0.001*) | 1.52 (1.28 – 1.81; <0.001*) | 190 / 705 (27.0%) | 1.30 (1.05 – 1.62; 0.016*) |

* Two-sided p-value is significant (p<0.05).

^a^ Adjusted for age, any medical condition, any mood stabilizer medication, current psychiatric disorder, any antidepressant, any mood stabilizer medication, and any antipsychotic medication.

Abbreviations: HR, hazard ratio; CI, confidence interval.

# Table S3. Associations between BZRA use and mortality in patients hospitalized for COVID-19, when including all patients who received a BZRA and considering BZRA use as a time-dependent variable.

|  | **Number of events / Number of patients** | **Crude Cox regression analysis** | **Multivariable Cox regression analysis** | **Analysis weighted by inverse-probability-weighting weights** | **Analysis weighted by inverse-probability-weighting weights adjusted for unbalanced covariates ^a^** | **Number of events / Number of patients in the matched groups** | **Crude Cox regression analysis** |
| --- | --- | --- | --- | --- | --- | --- | --- |
|  | **N (%)** | **HR (95% CI; p-value)** | **HR (95% CI; p-value)** | **HR (95% CI; p-value)** | **HR (95% CI; p-value)** | **N (%)** | **HR (95%CI;**  **p-value)** |
| No BZRA | 1,134 / 13,695 (8.3%) | Ref. | Ref. | Ref. | Ref. | 230 / 1,089 (21.1%) | Ref. |
| Any BZRA | 248 / 1,089 (22.8%) | 4.41 (4.11 – 5.41; <0.001*) | 2.59 (2.22 – 3.01; <0.001*) | 1.84 (1.53 – 2.23; <0.001*) | 1.55 (1.26 – 1.90; <0.001*) | 248 / 1,089 (22.8%) | 1.18 (0.87 – 1.60; 0.290) |

* Two-sided p-value is significant (p<0.05).

^a^ Adjusted for age, sex, hospital, medication according to compassionate use or as part of a clinical trial, any mood stabilizer medication, and any antipsychotic medication, any current psychiatric disorder and any mood stabilizer medication.

Abbreviations: HR, hazard ratio; CI, confidence interval.

# Table S4. Association between benzodiazepine receptor agonist (BZRA) use and the endpoint of death among patients who had been hospitalized for COVID-19 outside ICUs (N = 13,693).

|  | **Number of events / Number of patients** | **Crude Cox regression analysis** | **Multivariable Cox regression analysis** | **Analysis weighted by inverse-probability-weighting weights** | **Analysis weighted by inverse-probability-weighting weights adjusted for unbalanced covariates** | **Number of events / Number of patients in the matched groups** | **Crude Cox regression analysis** |
| --- | --- | --- | --- | --- | --- | --- | --- |
|  | **N (%)** | **HR (95%CI;**  **p-value)** | **HR (95%CI;**  **p-value) ^α^** | **HR (95%CI;**  **p-value) ^α^** | **HR (95%CI;**  **p-value) ^α^** | **N (%)** | **HR (95%CI;**  **p-value)** |
| No BZRA | 900 / 13,068 (6.9%) | Ref. | Ref. | Ref. | Ref. | 127 / 625 (20.3%) | Ref. |
| Any BZRA | 165 / 625 (26.4%) | 3.62 (2.72 – 4.80; <0.001*) | 2.10 (1.50 – 2.95; <0.001*) | 1.73 (1.39 – 2.15; <0.001*) | 1.73 (1.39 – 2.15; <0.001*) | 165 / 625 (26.4%) | 1.34 (1.06 – 1.69; 0.013*) |

* Two-sided p-value is significant (p<0.05).

Abbreviations: HR, hazard ratio; CI, confidence interval.

# Table S5. Association between benzodiazepine receptor agonist (BZRA) use at baesline and mortality, following additional adjustments for respiratory depression, any other clinical markers of disease severity, or both.

|  | **Number of events / Number of patients** | **Multivariable Cox regression analysis** | **Analysis weighted by inverse-probability-weighting weights** | **Analysis weighted by inverse-probability-weighting weights adjusted for unbalanced covariates** | **Number of events / Number of patients in the matched groups** | **Univariate Cox regression in a 1:1 ratio matched analytic sample** |
| --- | --- | --- | --- | --- | --- | --- |
|  | **N (%)** | **HR (95% CI; p-value)** | **HR (95% CI; p-value)** | **HR (95% CI; p-value)** | **N (%)** | **HR (95% CI; p-value)** |
| Main analyses without adjustments for respiratory depression or any other clinical markers of disease severity | | | | | | |
| No BZRA | 1,134 / 13,695 (8.3%) | Ref. | Ref. | Ref. | 143 / 686 (20.8%) | Ref. |
| Any BZRA | 186 / 686 (27.1%) | 1.94 (1.45 – 2.59; <0.001*) | 1.61 (1.31 – 1.98; <0.001*) | 1.56 (1.29 – 1.89; <0.001*) | 186 / 686 (27.1%) | 1.34 (1.08 – 1.67; 0.009*) |
| Analyses adjusting in addition for respiratory depression | | | | | | |
| No BZRA | 1,134 / 13,695 (8.3%) | Ref. | Ref. | Ref. | 144 / 686 (21.0%) | Ref. |
| Any BZRA | 186 / 686 (27.1%) | 1.86 (1.34 – 2.50; <0.001*) | 1.58 (1.29 – 1.94; <0.001*) | 1.57 (1.31 – 1.88; <0.001*) | 186 / 686 (27.1%) | 1.41 (1.14 – 1.76; 0.002*) |
| Analyses adjusting in addition for any other clinical markers of disease severity | | | | | | |
| No BZRA | 1,134 / 13,695 (8.3%) | Ref. | Ref. | Ref. | 140 / 686 (20.4%) | Ref. |
| Any BZRA | 186 / 686 (27.1%) | 1.83 (1.36 – 2.47; <0.001*) | 1.55 (1.27 – 1.90; <0.001*) | 1.54 (1.28 – 1.84; <0.001*) | 186 / 686 (27.1%) | 1.38 (1.11 – 1.72; 0.004*) |
| Analyses adjusting in addition for both respiratory depression and any other clinical markers of disease severity | | | | | | |
| No BZRA | 1,134 / 13,695 (8.3%) | Ref. | Ref. | Ref. | 136 / 686 (19.8%) | Ref. |
| Any BZRA | 186 / 686 (27.1%) | 1.83 (1.36 – 2.47; <0.001*) | 1.53 (1.25 – 1.88; <0.001*) | 1.52 (1.26 – 1.82; <0.001*) | 186 / 686 (27.1%) | 1.45 (1.16 – 1.81; 0.001*) |

* Two-sided p-value is significant (p<0.05).

Respiratory depression was defined by a respiratory rate < 12 breaths/min or a resting peripheral capillary oxygen saturation in ambient air < 90%; any other clinical markers of disease severity was defined by a temperature > 40°C or a systolic blood pressure < 100 mmHg or a respiratory rate > 24 breaths/min or a plasma lactate levels higher than 2 mmol/L.

Abbreviations: HR, hazard ratio; CI, confidence interval.

**Table S6. Associations between individual benzodiazepine receptor agonists (BZRAs) and mortality.**

|  | **Number of events / Number of patients** | **Crude Cox regression analysis** | **Multivariable Cox regression analysis** | **Analysis weighted by inverse-probability-weighting weights** | **Analysis weighted by inverse-probability-weighting weights adjusted for unbalanced covariates** |
| --- | --- | --- | --- | --- | --- |
|  | **N (%)** | **HR (95%CI; p-value)** | **HR (95%CI; p-value)** | **HR (95%CI; p-value)** | **HR (95%CI; p-value)** |
| No BZRA | 1,134 / 13,695 (8.3) | Ref. | Ref. | Ref. | Ref. |
| Diazepam | 9 / 74 (12.2) | 1.48 (0.77 - 2.86; 0.240) | 0.85 (0.40 – 1.82; 0.678) | 0.84 (0.29 – 2.46; 0.747) | 1.20 (0.56 – 2.60; 0.636) |
| Any BZRA other than diazepam | 177 / 612 (28.9) | 3.39 (2.90 - 3.98; <0.001*) | 2.02 (1.50 – 2.73; <0.001*) | 1.63 (1.33 – 2.01; <0.001*) | 1.68 (1.40 – 2.01; <0.001*) |
| Alprazolam | 32 / 129 (24.8) | 2.56 (1.80 – 3.63; <0.001*) | 1.19 (0.82 – 1.72; 0.366) | 1.22 (0.79 – 1.89; 0.359) | 1.23 (0.82 – 1.85; 0.987) |
| Clonazepam | 9 / 43 (20.9) | 2.19 (1.14 – 4.22; 0.019*) | 1.28 (0.65 – 2.50; 0.476) | 1.32 (0.65 – 2.69; 0.442) | 2.55 (0.98 – 6.65; 0.055) |
| Midazolam | 74 / 125 (59.2) | 8.34 (6.59 – 10.57; <0.001*) | 3.72 (2.90 – 4.78; <0.001*) | 3.02 (2.29 – 3.99; <0.001*) | 3.06 (2.35 – 4.01; <0.001*) |
| Oxazepam | 57 / 234 (24.4) | 3.05 (1.97 – 4.72; <0.001*) | 1.59 (0.96 – 2.61; 0.070) | 1.64 (1.16 – 2.31; 0.005*) | 1.44 (1.06 – 1.97; 0.022*) |
| Z-drugs (zopiclone or zolpidem) | 42 / 195 (21.5) | 3.08 (1.89 – 5.03; <0.001*) | 1.79 (0.98 – 3.24; 0.057) | 1.98 (1.22 – 3.22; 0.006*) | 1.39 (0.96 – 2.00; 0.081) |
| Other BZRAs | 23 / 129 (17.8) | 1.95 (1.29 – 2.95; 0.002*) | 1.12 (0.66 – 1.90; 0.688) | 1.72 (0.92 – 3.20; 0.089) | 1.27 (0.63 – 2.54; 0.500) |
| Any benzodiazepine receptor agonist other than diazepam or midazolam | 104 / 494 (21.1) | 2.76 (1.99 – 3.83; <0.001*) | 1.60 (1.09 – 2.35; 0.018*) | 2.13 (1.52 – 2.98; <0.001*) | 1.71 (1.22 – 2.38; 0.002*) |

* Two-sided p-value is significant (p<0.05).

Abbreviations: HR, hazard ratio; CI, confidence interval.

Only individual BZRAs associated with more than 5 end-point events are presented in the table.

# Table S7. Characteristics of patients with COVID-19 receiving diazepam versus any other benzodiazepine receptor agonist (BZRA) (N=686).

|  | **Diazepam**  **(N = 74)** | **Other benzodiazepine receptor agonists**  **(N = 612)** | **Non-exposed matched group**  **(N=74)** | **Diazepam**  **vs.**  **Other benzodiazepine receptor agonists** | **Diazepam vs.**  **Other benzodiazepine receptor agonists** | **Diazepam vs.**  **Non-exposed matched group** |
| --- | --- | --- | --- | --- | --- | --- |
|  |  |  |  | **Crude analysis** | **Analysis weighted by inverse-probability-weighting weights** | **Matched analytic sample analysis** |
|  | **N (%)** | **N (%)** | **N (%)** | **SMD** | **SMD** | **SMD** |
| Age |  |  |  | 0.592 | 0.140 | 0.276 |
| *18 to 50 years* | 13 (17.6%) | 57 (9.31%) | 10 (13.5%) |  |  |  |
| *51 to 70 years* | 34 (45.9%) | 158 (25.8%) | 27 (36.5%) |  |  |  |
| *More than 70 years* | 27 (36.5%) | 397 (64.9%) | 37 (50.0%) |  |  |  |
| Sex |  |  |  | 0.458 | 0.091 | 0.114 |
| *Women* | 23 (31.1%) | 325 (53.1%) | 27 (36.5%) |  |  |  |
| *Men* | 51 (68.9%) | 287 (46.9%) | 47 (63.5%) |  |  |  |
| Hospital |  |  |  | 0.253 | 0.136 | 0.073 |
| *AP-HP Centre – Paris University, Henri Mondor University Hospitals and at home hospitalization* | 26 (35.1%) | 180 (29.4%) | 28 (37.8%) |  |  |  |
| *AP-HP Nord and Hôpitaux Universitaires Paris Seine-Saint-Denis* | 21 (28.4%) | 201 (32.8%) | 21 (28.4%) |  |  |  |
| *AP-HP Paris Saclay University* | 17 (23.0%) | 105 (17.2%) | 15 (20.3%) |  |  |  |
| *AP-HP Sorbonne University* | 10 (13.5%) | 126 (20.6%) | 10 (13.5%) |  |  |  |
| Obesity ^α^ |  |  |  | 0.091 | 0.024 | 0.135 |
| *Yes* | 17 (23.0%) | 118 (19.3%) | 13 (17.6%) |  |  |  |
| *No* | 57 (77.0%) | 494 (80.7%) | 61 (82.4%) |  |  |  |
| Smoking ^β^ |  |  |  | 0.115 | 0.046 | 0.221 |
| *Yes* | 15 (20.3%) | 97 (15.8%) | 9 (12.2%) |  |  |  |
| *No* | 59 (79.7%) | 515 (84.2%) | 65 (87.8%) |  |  |  |
| Any medical condition ^γ^ |  |  |  | 0.257 | 0.043 | 0.027 |
| *Yes* | 34 (45.9%) | 359 (58.7%) | 35 (47.3%) |  |  |  |
| *No* | 40 (54.1%) | 253 (41.3%) | 39 (52.7%) |  |  |  |
| Medication according to compassionate use or as part of a clinical trial ^ϴ^ |  |  |  | 0.012 | 0.032 | 0.064 |
| *Yes* | 18 (24.3%) | 152 (24.8%) | 16 (21.6%) |  |  |  |
| *No* | 56 (75.7%) | 460 (75.2%) | 58 (78.4%) |  |  |  |
| Any current psychiatric disorder ^¥^ |  |  |  | 0.288 | 0.138 | 0.274 |
| *Yes* | 25 (33.8%) | 129 (21.1%) | 16 (21.6%) |  |  |  |
| *No* | 49 (66.2%) | 483 (78.9%) | 58 (78.4%) |  |  |  |
| Any antidepressant |  |  |  | 0.346 | 0.046 | 0.031 |
| *Yes* | 20 (27.0%) | 265 (43.3%) | 19 (25.7%) |  |  |  |
| *No* | 54 (73.0%) | 347 (56.7%) | 55 (74.3%) |  |  |  |
| Any mood stabilizer medication ^Ω^ |  |  |  | 0.129 | 0.094 | 0.096 |
| *Yes* | 19 (25.7%) | 124 (20.3%) | 16 (21.6%) |  |  |  |
| *No* | 55 (74.3%) | 488 (79.7%) | 58 (78.4%) |  |  |  |
| Any antipsychotic medication |  |  |  | 0.647 | 0.220 | 0.333 |
| *Yes* | 37 (50.0%) | 126 (20.6%) | 25 (33.8%) |  |  |  |
| *No* | 37 (50.0%) | 486 (79.4%) | 49 (66.2%) |  |  |  |
| Number of BZRA medications |  |  |  | 0.325 | 0.084 | 0.124 |
| *1* | 45 (60.8%) | 458 (74.8%) | 49 (66.2%) |  |  |  |
| *2* | 22 (29.7%) | 130 (21.2%) | 18 (24.3%) |  |  |  |
| *3 or more* | 7 (9.46%) | 24 (3.92%) | 7 (9.46%) |  |  |  |

^α^ Defined as having a body-mass index higher than 30 kg/m^2^ or an International Statistical Classification of Diseases and Related Health Problems (ICD-10) diagnosis code for obesity (E66.0, E66.1, E66.2, E66.8, E66.9).

^β^ Current Smoking status was self-reported.

^γ^ Assessed using ICD-10 diagnosis codes for diabetes mellitus (E11), diseases of the circulatory system (I00-I99), diseases of the respiratory system (J00-J99), neoplasms (C00-D49), diseases of the blood and blood-forming organs and certain disorders involving the immune mechanism (D5-D8), frontotemporal dementia (G31.0), peptic ulcer (K27), diseases of liver (K70-K95), hemiplegia or paraplegia (G81-G82), acute kidney failure or chronic kidney disease (N17-N19), and HIV (B20).

^ϴ^ Any medication prescribed as part of a clinical trial or according to compassionate use (e.g., hydroxychloroquine, azithromycin, remdesivir, tocilizumab, sarilumab, or dexamethasone).

^¥^ Assessed using ICD-10 diagnosis codes (F00- F99).

^Ω^ Included lithium or antiepileptic medications with mood stabilizing properties.

SMD>0.1 indicates substantial difference.

Abbreviation: SMD, standardized mean difference.

# Table S8. Association between diazepam use and the endpoint of death among patients who had been hospitalized for COVID-19 and had received benzodiazepine receptor agonists at baseline (N=686).

|  | **Number of events / Number of patients** | **Crude Cox regression analysis** | **Multivariable Cox regression analysis** ^a^ | **Analysis weighted by inverse-probability-weighting weights** | **Analysis weighted by inverse-probability-weighting weights adjusting for unbalanced covariates** ^b^ | **Number of events /**  **Number of patients in the matched groups** | **Univariate Cox regression in the 1:1 ratio matched analytic sample** | **Univariate Cox regression in the 1:1 ratio matched analytic sample adjusting for unbalanced covariates**^c^ |
| --- | --- | --- | --- | --- | --- | --- | --- | --- |
|  | N (%) | HR (95%CI; p-value) | HR (95%CI; p-value) | HR (95%CI; p-value) | HR (95%CI; p-value) | N (%) | HR (95%CI;  p-value) | HR (95%CI;  p-value) |
| Diazepam | 9 / 74 (12.2) | 0.44 (0.23 – 0.86; 0.017*) | 0.49 (0.24 – 0.98; 0.044*) | 0.31 (0.13 – 0.74; 0.008*) | 0.37 (0.15 – 0.91; 0.029*) | 9 / 74 (12.2) | 0.43 (0.20 - 0.95; 0.036*) | 0.36 (0.14 – 0.96; 0.041*) |
| Other benzodiazepine receptor agonists | 177 / 612 (28.9) | Ref. | Ref. | Ref. | Ref. | 21 / 74 (28.4) | Ref. | Ref. |

* Two-sided p-value is significant (p<0.05).

^a^ Adjusted for sex, age, hospital, obesity, current smoking status, any significant medical, any current psychiatric disorder, any medication prescribed according to compassionate use or as part of a clinical trial, other psychotropic medications (i.e. any antidepressant, any mood stabilizer and any antipsychotic medication), and number of BZRA medications.

^b^ Adjusted for age, hospital, any current psychiatric disorder, and any antipsychotic medication.

^c^ Adjusted for age, sex, obesity, smoking, any current psychiatric disorder, any antipsychotic medication, and number of BZRA medications.

Abbreviations: HR, hazard ratio; CI, confidence interval.
